# Diagnostic accuracy of resting systolic toe pressure for diagnosis of peripheral arterial disease in people with and without diabetes: a cross-sectional retrospective case-control study

**DOI:** 10.1186/s13047-017-0236-z

**Published:** 2017-12-19

**Authors:** Peta Ellen Tehan, Alex Louise Barwick, Mathew Sebastian, Vivienne Helaine Chuter

**Affiliations:** 1 School of Health Sciences, Faculty of Health and Medicine, University of Newcastle, Ourimbah, NSW 2258 Australia; 2 Priority Research Centre for Generational Health and Ageing, Hunter Medical Research Institute, New Lambton Heights, Australia; 3 School of Health and Human Sciences, Southern Cross University, Gold Coast, Qld Australia; 4 Vascular Health Care, Lake Macquarie, NSW Australia; 5 Department of Surgery, John Hunter Hospital, New Lambton, NSW Australia

**Keywords:** Toe pressure, Sensitivity, Specificity, Peripheral arterial disease

## Abstract

**Background:**

The resting systolic toe pressure (TP) is a measure of small arterial function in the periphery. TP is used in addition to the ankle-brachial index when screening for peripheral arterial disease (PAD) of the lower limb in those with diabetes, particularly in the presence of lower limb medial arterial calcification. It may be used as an adjunct assessment of lower limb vascular function and as a predictor of wound healing. The aim of this study was to determine the diagnostic accuracy of TP for detecting PAD in people with and without diabetes.

**Methods:**

This was a retrospective case-control study. Two researchers extracted information from consecutive patient records, including TP measurements, colour Duplex ultrasound results, demographic information, and medical history. Measures of diagnostic accuracy were determined by receiver operating curve (ROC) analysis, and calculation of sensitivity, specificity, and positive and negative likelihood ratios.

**Results:**

Three hundred and nintey-four participants with suspected PAD were included. In the diabetes group (*n* = 176), ROC analysis of TP for detecting PAD was 0.78 (95%CI: 0.69 to 0.84). In the control group (*n* = 218), the ROC of TP was 0.73 (95%CI: 0.70 to 0.80). TP had highest sensitivity when anatomical distribution of disease was both proximal and distal (diabetes group: 79.49%, the control group: 82.61%). TP yielded highest sensitivity in mild disease (50–75% stenosis) in diabetes group, (81.82%) and moderate disease (>75% stenosis) in control group (80.77%).

**Conclusions:**

Our findings indicate that TPs are useful to assist in diagnosing PAD in clinical practice, however, results should be interpreted with caution due to the small probability of PAD being present with a negative test.

## Background

The early diagnosis and treatment of peripheral arterial disease (PAD) in the lower limb requires effective clinical assessment of peripheral vascular status [1]. The presence of a number of factors including diabetes, renal disease and older age are known to affect the accuracy of currently recommended large artery assessment methods such as the ankle-brachial index (ABI) [2–4]. In those with diabetes, there is substantial evidence that the ABI has reduced diagnostic accuracy for identification of lower limb vascular disease, particularly in early stages of the disease [5]. This limited accuracy is attributed to the increased likelihood of co-existent vascular complications including medial arterial wall calcification (MAC) and a more distal anatomical distribution of disease [2, 6–8].

The resting systolic toe pressure (TP) is a measure of small arterial function in the periphery. TP is a reliable [9, 10], non-invasive, time and cost-efficient tool. It is currently used as an adjunct to the ABI when screening for PAD of the lower limb in those with diabetes, particularly in the presence of suspected lower limb MAC (ABI result exceeding 1.3 or 1.4) [5, 11]. It is also used as part of an objective assessment of lower limb vascular function when used in calculation of the toe brachial index (TBI)- a ratio of TP to brachial pressure [12]. Low TP has also been demonstrated to be predictive of foot ulcer healing outcomes, with a TP of <30 mmHg found to increase the likelihood of non-healing of lower limb ulcers by more than three-fold in a majority diabetes cohort [13]. This is supported by current recommendations of the International Working Group on the Diabetic Foot for urgent imaging and revascularisation in those with a diabetes-related foot ulcer and TP of <30 mmHg [14].

Although TP have been shown to have predictive capacity for wound healing capacity, which is generally multifactorial, there has been little investigation of the diagnostic accuracy of this test for identification of PAD, which may be present without an active wound. The TBI has recently been shown to have superior diagnostic accuracy to the ABI for identifying PAD in a community-based diabetes cohort [7] suggesting TP may also be a useful diagnostic tool for this purpose, while requiring less clinical time to perform. The aims of this study were firstly, to determine the diagnostic accuracy of TP for detecting haemodynamically significant PAD and, secondly, to determine the optimum diagnostic threshold for diagnosis of PAD, in people with and without diabetes. Finally, we aimed to investigate the effect of anatomical location and severity of disease on the sensitivity of TP for detecting PAD in people with and without diabetes.

### Methods

This retrospective, case-control study was undertaken at Vascular Health Care, a private vascular laboratory in Lake Macquarie, New South Wales, Australia. Ethics approval was obtained from the University of Newcastle Human Research Ethics Committee (H2010–1230). Data were extracted by two researchers (PT and AB) from consecutive medical records of patients referred to the vascular laboratory for investigations due to suspected PAD. Only data from patients who had both TP and colour Duplex ultrasound (CDU) testing performed were included. Extracted data included demographic information and relevant medical history e.g. age, gender, diabetes status, smoking status and history of foot complications. Information on the presence and extent of stenosis, MAC and recorded signs and symptoms of intermittent claudication were also extracted.

All data from vascular investigations were extracted from testing sessions attended by the participant at the vascular clinic from one single limb. The right limb was chosen when both limb data were available. The vascular investigations had all been completed by a vascular ultrasonographer in accordance with standard clinical protocols. These include patients being rested in a supine position for ten minutes prior to TP measurements being undertaken in a temperature controlled room (23–25 °C) [15]. Participants are asked to avoid alcohol, smoking, exercise and caffeine one hour prior to the testing session to avoid influencing pressure measurement, and, are fasted for 12 h prior to the testing session to enable adequate visualisation of vessels in the abdomen without interference from gas [16].

Toe pressures are taken using the Parks Vascular Mini Lab 1050c, photoplethysmography probe (PPG) with 1.6, 1.9 or 2.5 cm inflatable cuff and ERKA switch blood pressure gauge. The size of the cuff used is in accordance with current guidelines for cuff size [11]. The photoplethesmography (PPG) probe is placed on the distal pulp of the hallux or second toe and affixed with micropore tape. The cuff is placed directly proximal to the probe. Once a regular signal from the PPG probe is obtained, the toe cuff is inflated 20 mmHg above loss of the signal. The cuff is then slowly deflated at a rate of 2 mmHg per second. The first regular upstroke of the PPG signal is considered the systolic toe pressure.

CDU is then performed on the lower limb from the distal aorta to the foot and for this study this was used as the reference standard [17, 18]. A Phillips CX-50 or GE Logiq-I is used to complete CDU. Grading of stenosis is conducted in accordance with the following criteria: >50–75% stenosis (>50%): focal increase in velocities 250 cm/s-350 cm/s and greater than threefold increase in velocities, 75–99% (>75%) stenosis- focal increase in velocities >350 cm/s, fourfold increase in velocities, occlusion- vessel well visualised, no colour or Doppler flow seen [19, 20]. The reliability CDU and pressure testing performed by this vascular laboratory has previously been shown to be acceptable [21].

Sensitivity of TP for detecting PAD was examined by anatomical location of disease and disease severity. For anatomical location of disease, participant data were grouped into those with proximal disease only, those with distal disease only and those with both proximal and distal disease. Proximal disease was defined as disease from the common iliac artery to the distal superficial femoral artery, and distal disease was disease distal to, and including, the proximal popliteal artery. Sensitivity was also determined for severity of disease, with participant data grouped by disease severity of the worst affected vessel and classified as mild (>50–75% stenosis), moderate (>75–99% stenosis) and occlusive disease (occlusion).

To compare TP between the participant groups with diabetes (diabetes group) and without diabetes (control group), an independent samples t-test was performed. Pearson’s chi-square was performed to compare between group differences for gender, history of smoking and rates of PAD (present or absent). Statistical significance was set at *p* < 0.05.

For the purposes of reporting and statistical analyses, presence of PAD was defined as at least one arterial stenosis of >50% of the lumen diameter, as determined by CDU, being present in the limb tested [19, 20, 22]. Receiver Operating Characteristic (ROC) analyses were performed to determine the performance of TP for the diagnosis of CDU-diagnosed PAD for people with and without diabetes and areas under the curve (AUC) calculated. AUCs were interpreted in accordance with Portney and Watkins (2009) with 0.90 to 1.0 an excellent test, 0.80 to 0.90 a good test, 0.70 to 0.80 a fair test 0.60 to 0.70 a poor test and 0.50 to 0.60 a failed test [23]. All analyses were conducted using SPSS version 22 statistical software. Sensitivity, specificity, positive and negative likelihood ratios with 95% confidence intervals were all calculated in Microsoft Excel.

### Results

A total of 394 participants were included, 176 with diabetes (diabetes group) and 218 without diabetes (control group). Participant demographics are shown in Table 1, along with comparative statistics which showed no significant between group differences for gender, smoking history, rates of PAD or mean TP. Mean TP was 90 mmHg in the diabetes group, and 87 mmHg in the control group.

**Table 1:** Participant Characteristics

	DM Group	Control Group	Comparison
Total Limbs N	176	218	
Males n (%)	115 (65)	125 (57)	^B^ (*p* = 0.12)
Females n (%)	62 (35)	93 (43)	
Age Range (Years)	53–94	56–95	
Mean Age (years)(^A^)	74.60 (8.39)	78.33 (8.59)	
History of foot complications	13 (7)	20 (9)	
History of smoking (%)	103 (58)	110 (40)	^B^ (*p* = 0.13)
Currently smoking (%)	9 (5)	16 (7)	
Mean systolic TP (^A^)	90.43 (35.10)	87.59 (34.10)	(*p* = 0.42)
Calcification visualised on CDU (%)	49 (27)	85 (38)	
Distal PAD n (%)	61 (34)	82 (37)	
Proximal PAD n (%)	18 (9)	33 (15)	
Distal and Proximal PAD n (%)	39 (22)	46 (21)	
PAD prevalence n (%)	118 (67)	162 (74)	^**B**^ (*p* = 0.11)
>50% stenosis n (%)	11(6)	18 (6)	
>75% stenosis n (%)	11 (6)	26 (11)	
Occlusion n (%)	96 (55)	117 (55)	

^A^=standard deviation, PAD = peripheral arterial disease, ^B^Pearson’s chi-square

**Fig. 1:**
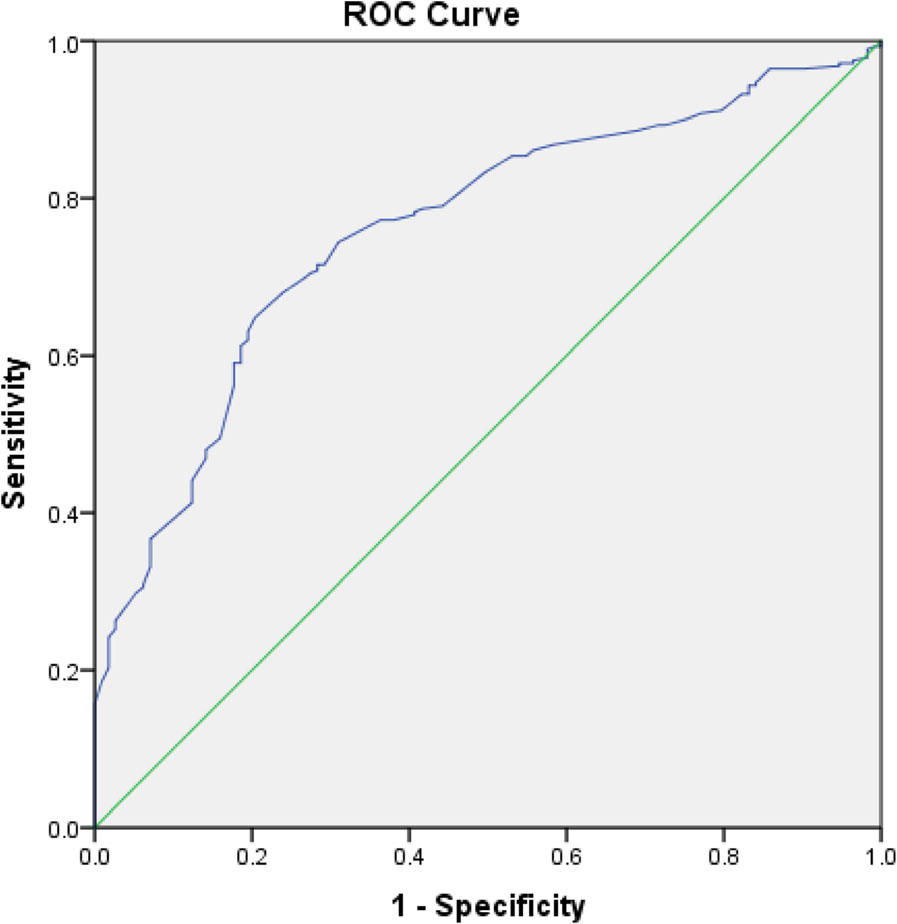
ROC analysis of TP for detecting PAD in the whole population

**Fig. 2:**
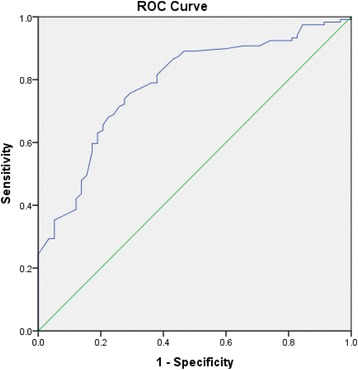
ROC analysis of TP for detecting PAD in people with diabetes

**Fig. 3:**
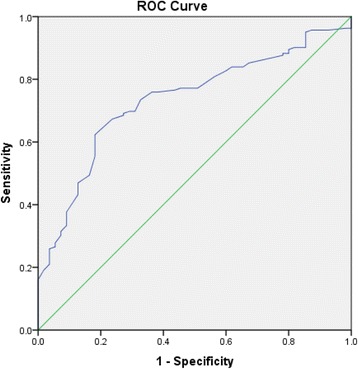
ROC analysis of TP for detecting PAD in people without diabetes

In the combined group analysis (*N* = 394 ) the ROC of TP for detecting PAD had an AUC of 0.76 (95% CI: 0.66 to 0.80) (Fig. 1), with an optimum diagnostic threshold of 96.5 mmHg. In the diabetes group, the ROC AUC for TP was 0.78 (95% CI: 0.71 to 0.85) (Fig. 2), with an optimum diagnostic threshold of 97 mmHg. Sensitivity for TP for detecting PAD in the diabetes group was 73.73% (95% CI: 64.30 to 81.40) and specificity was 72.41% (95% CI:59.10 to 83.84) (Table 2). In the control group, the ROC AUC for TP was 0.73 (95% CI: 0.66 to 0.80) (Fig. 3) with an optimum diagnostic threshold of 96 mmHg. Sensitivity of TP for detecting PAD was 67.26% (95% CI: 59.61 to 74.29) (Table 2) and specificity was 71.43% (95% CI: 57.79 to 82.70).

**Table 2:** Diagnostic Accuracy of TP for detecting PAD in diabetes and control groups

	*Diabetes Group (N = 176)*	*Control Group (N =218 )*
	*Systolic Toe Pressure(95% CI)*	*Systolic Toe pressure (95% CI)*
*Sensitivity*	73.73 (64.30 to 81.40)	67.26 (59.61 to 74.29)
*Specificity*	72.41 (59.10 to 83.84)	71.43 (57.79 to 82.70)
*Positive Likelihood Ratio*	2.67^a^ (1.74 to 4.11)	2.35^a^ (1.54 to 3.61)
*Negative Likelihood Ratio*	0.36^a^ (0.26 to 0.51)	0.46^a^ (0.35 to 0.60)

^a^may be important effect

Sensitivity of TP for detecting PAD by anatomical location of PAD (Table 3) showed TP had the highest sensitivity in those with both proximal and distal disease for the diabetes group (79.49%, 95% CI: 63.54 to 90.70), and control group (82.61%, 95% CI: 68.58 to 92.18). The sensitivity of TP according to severity of disease (Table 3) found that TP had highest sensitivity for mild disease in the diabetes group (81.82%, 95% CI: 48.22 to 97.72) and for moderate disease in the control group (80.77%, 95% CI: 60.65 to 93.45). Specificity of TP for detecting PAD was similar in both groups, with 72.41% (95% CI: 59.10 to 83.84) for the diabetes group and 71.43% (95% CI: 57.79 to 82.70) for the control group.

**Table 3:** Sensitivity of TP according to disease location and severity

	*Participants with Diabetes (N = 176)*		*Control Group (N = 218)*	
PAD anatomical distribution	*N*	TP sensitivity (95%CI)	N	TP sensitivity (95%CI)
* Proximal disease*	18	77.78 (52.36 to 93.59)	33	69.70 (51.20 to 84.41)
* Distal disease*	61	68.85 (55.71 to 80.10)	82	61.73 (50.26 to 72.31)
* Both proximal and distal disease*	39	79.49 (63.54 to 90.70)	46	82.61 (68.58 to 92.18)
PAD severity	N	TP sensitivity (95%CI)	N	TP sensitivity (95%CI)
* >50% - 75%*	11	81.82 (48.22 to 97.72)	18	50.00 (26.02 to 73.98)
* >75%*	11	45.45 (16.75 to 76.62)	26	80.77 (60.65 to 93.45)
* Occlusion*	96	76.04 (66.25 to 84.17)	117	70.94 (61.83 to 78.96)

### Discussion

Whilst TP are used frequently by vascular laboratories, podiatrists and physicians, particularly in people with diabetes, the evidence for the diagnostic accuracy and appropriate diagnostic thresholds of this testing method for detecting PAD is lacking. To our knowledge, this is the first substantial study evaluating the diagnostic accuracy of TP compared to diagnostic imaging for diagnosing PAD in cohorts with suspected PAD, with and without diabetes.

This study found TP to be a fair test for detecting PAD in a mixed population (with and without diabetes) with suspected PAD (ROC AUC 0.76) [24]. This was also the case in subanalysis of people with diabetes and suspected PAD (ROC AUC 0.78) and in a control group of people without diabetes with suspected PAD (ROC AUC 0.73) [23]. Of note, presence of diabetes did not appear to affect the diagnostic accuracy of PAD. In contrast, previous studies have demonstrated the diagnostic accuracy of multiple non-invasive testing methods to be reduced in diabetes cohorts when compared to cohorts without diabetes [7, 8]. The findings of our present study suggest that TP may be less affected by vascular complications associated with diabetes which are known to reduce accuracy of large artery testing methods, such as the presence of MAC. This would, to some extent, explain the similar performance of the test in both groups. However, as we did not compare the diagnostic accuracy of TP to the results of other methods of lower limb vascular assessment in these participants, it is also possible that improvement in test performance in the diabetes group was associated with the particular participants included in this study i.e. those with suspected PAD. As over 50% of the participants in both groups had occlusions, and there was a high overall prevalence of PAD of >50% (67% diabetes group, 74% control group), there is a strong likelihood that the test performance was overestimated for both groups, limiting the generalisability of these findings. Nevertheless, our results do support the role of TP to assist with diagnosis of PAD where there is clinical suspicion of the disease in either population.

The current understanding of the clinical utility of TP is limited by a lack of evidence relating to normal values and thresholds that are indicative of PAD specifically. Previous research has focused primarily on wound healing outcomes with TP thresholds between 30 mmHg and 70 mmHg identified as predicting likelihood of wound healing [25]. As part of this investigation, we identified diagnostic thresholds of TP for PAD in people with and without diabetes by ROC analysis. Notably the thresholds for PAD (>50% stenosis) were similar in the group with diabetes (<97 mmHg) and the control group (<96 mmHg), and significantly higher than previously identified thresholds for wound healing [13]. Given the severity of PAD in the majority of participants in this study was high, (>50% with occlusive disease), this finding suggests significant PAD is likely to be present in people with TP well in excess of previously identified critical thresholds [25], and, contributes substantially to current understanding of the clinical utility of this clinical test. Both severity and anatomical distribution of disease affected the sensitivity of TP for detecting PAD. Relatively low numbers of participants with mild and moderate disease in both groups means these results need to be interpreted with caution, our initial findings warrant further large-scale investigation. This is of particular relevance to the diabetes group were there were only 11 participants in each of the >50 to 75% and the >75% categories. In this group, TP was more sensitive in mild disease (>50–75% stenosis) suggesting this test may play a particularly valuable role for early identification of disease. This, in conjunction with our findings that TP thresholds for diagnosis of PAD are higher than those previously identified for wound healing, highlights a role for clinical lower limb vascular assessment in early diagnosis of PAD. TP may significantly contribute to a clinician’s ability to identify presence of PAD early in the disease process, allowing for effective management of cardiovascular risk factors to help prevent disease progression and associated adverse cardiovascular and cerebrovascular outcomes [25]. Furthermore, while the ABI has been demonstrated to have high specificity in people with diabetes, sensitivity has been shown to be low, suggesting less capacity for early disease detection [2, 7, 8].

When analysing diagnostic accuracy of TP by anatomical location of disease, the highest sensitivity occurred when disease was located both proximally and distally in both the diabetes and control groups. This diffuse anatomical disease distribution most likely led to a cumulative, significant, pressure drop distally. TP yielded the lowest sensitivity in both groups when disease was distally anatomically located only. Distal disease is frequently reported to negatively impact accuracy of non-invasive testing methods, particularly in diabetes cohorts [2, 7], due to the co-existence of MAC and occlusive PAD in extended vessel segments [26]. This has implications for vascular testing in both diabetes and older cohorts. In these populations it is established that there is a predilection for more distally located lesions [2, 6]. Our study results demonstrate this pattern of disease does not result in the same pressure reduction at the hallux as a more diffuse proximal and distal PAD presentation. These findings highlight the need for a multifaceted approach to PAD diagnosis rather than reliance of one objective measurement. In both groups, positive likelihood ratios and negative likelihood ratios were interpreted as potentially important, indicating that PAD was likely to be present with a positive test with a small probability of PAD being present with a negative test. This suggests that TP are a fair test for use in clinical practice, however should not be used as a standalone test due to the small probability of PAD being present with a normal pressure reading.

The findings of this study should also be interpreted in light of some potential limitations not previously discussed. Due to the retrospective nature of the study, we were not able to blind the vascular sonographers to the results of the index test, prior to completion of the reference standard. The reference standard used was CDU, which is a valid imaging technique and is used extensively clinically, however, angiography remains the gold standard for diagnosis of PAD. Our population was selected from those referred for vascular testing and therefore was more diseased than the general population (67% DM, 74% control). As previously stated this is likely to have caused some degree of spectrum bias and overestimation of the diagnostic accuracy of the test [27]. In addition it also prevented the calculation of positive and negative predictive values [28]. Our population was also older, and had a large proportion of males, however, this is distribution is more typical of the population presenting to a vascular clinic for screening [29]. Both the control and diabetes groups has significant rates of MAC visualised on CDU. It is possible that MAC may have affected digital arteries in some participants and elevated the TP. As there is currently no established upper limit for a normal TP, it is not possible for the likelihood of this to be accurately estimated.

### Conclusion

In conclusion, TPs are a useful adjunct test for non-invasive vascular assessment of lower limb. Results should be interpreted in conjunction with other vascular assessment results due to some risk of false positive and false negative results. TP values of <97 mmHg in people with diabetes and <96 mmHg in those without diabetes are indicative of PAD and the test has acceptable diagnostic accuracy in both cohorts. Our initial findings also suggest that TP may be effective in detection of less severe PAD, however, further larger scale investigation is required to confirm this.
